# Autistic individuals benefit from gestures during degraded speech comprehension

**DOI:** 10.1177/13623613241286570

**Published:** 2024-10-05

**Authors:** Sara Mazzini, Noor Seijdel, Linda Drijvers

**Affiliations:** 1 Max Planck Institute for Psycholinguistics, Nijmegen, The Netherlands; 2 Radboud University, Donders Institute for Brain, Cognition and Behaviour, Nijmegen, The Netherlands

**Keywords:** autism, degraded speech comprehension, multimodal language, multisensory integration, speech-gesture integration

## Abstract

Meaningful gestures enhance degraded speech comprehension in neurotypical adults, but it is unknown whether this is the case for neurodivergent populations, such as autistic individuals. Previous research demonstrated atypical multisensory and speech-gesture integration in autistic individuals, suggesting that integrating speech and gestures may be more challenging and less beneficial for speech comprehension in adverse listening conditions in comparison to neurotypicals. Conversely, autistic individuals could also benefit from additional cues to comprehend speech in noise, as they encounter difficulties in filtering relevant information from noise. We here investigated whether gestural enhancement of degraded speech comprehension differs for neurotypical (*n* = 40, mean age = 24.1) compared to autistic (n = 40, mean age = 26.8) adults. Participants watched videos of an actress uttering a Dutch action verb in clear or degraded speech accompanied with or without a gesture, and completed a free-recall task. Gestural enhancement was observed for both autistic and neurotypical individuals, and did not differ between groups. In contrast to previous literature, our results demonstrate that autistic individuals do benefit from gestures during degraded speech comprehension, similar to neurotypicals. These findings provide relevant insights to improve communication practices with autistic individuals and to develop new interventions for speech comprehension.

**Lay Abstract:**

Our study explored how meaningful hand gestures, alongside spoken words, can help autistic individuals to understand speech, especially when the speech quality is poor, such as when there is a lot of noise around. Previous research has suggested that meaningful hand gestures might be processed differently in autistic individuals, and we therefore expected that these hand gestures might aid them less in understanding speech in adverse listening conditions than for non-autistic people. To this end, we asked participants to watch and listen to videos of a woman uttering a Dutch action verb. In these videos, she either made a meaningful gesture while speaking, or not, and speech was clear, or noisy. The task for participants was to identify the verb in the videos. Contrary to what we expected, we found that both autistic and non-autistic individuals use meaningful information from hand gestures when understanding unclear speech. This means that gestural information can aid in communication, especially when communicative settings are suboptimal.

## Introduction

During face-to-face communication, interlocutors must integrate speech and visual bodily signals, such as hand gestures. Specifically iconic gestures, which can be described as hand gestures that illustrate object attributes, actions, and space (McNeill, 1992), have been demonstrated to improve speech comprehension in adverse listening conditions (in both multi-talker babble and noise-vocoded (hereafter: degraded) speech, see, e.g., Drijvers & Özyürek, 2017). However, this effect has not yet been investigated in autistic individuals, who commonly encounter socio-communicative differences. We here fill this gap in the literature.

Social communication differences represent one of the behavioral phenotypes of autism (Hodges et al., 2020), and altered multisensory integration has been observed in autism for both speech (Foxe et al., 2015; Smith & Bennetto, 2007) and non-speech stimuli (i.e., flashes and tones, see Beker et al., 2018). Only a few studies have investigated speech-gesture integration in autism. Trujillo et al. (2021) reported that autistic individuals were overall unimpaired when recognizing gestures, but they interpret more complex movements differently than neurotypical adults do. Moreover, Silverman et al. (2010) demonstrated that autistic children and adolescents were slower to respond to audiovisual stimuli than to audio-only stimuli and that gestures did not increase accuracy, which suggests that hand gestures may hinder, and not benefit, speech comprehension.

There are a few theoretical explanations of why speech-gesture integration might work differently in autism: The Weak Central Coherence theory (Happé & Frith, 2006) posits that a focus on local details might hinder global integration. Moreover, the Enhanced Perceptual Functioning theory (Mottron et al., 2006) suggests that autistic individuals possess heightened perceptual capacities for low-level information (e.g., detection, discrimination and categorization of perceptual stimuli), which causes the weighting of that information to be stronger than higher-order information, such as semantic information conveyed by iconic gestures. Both theories would predict that gestures might not aid speech comprehension for autistic individuals in adverse listening conditions, as they might focus more on low-level, local information, which might hinder integration. In fact, these theories would suggest that autistic individuals mostly have difficulty processing the degraded speech, as they might focus on the low-level alterations in the speech signal more.

In the present study, we therefore directly tested gestural enhancement of degraded speech in neurotypical and autistic adults, to better understand how autistic adults make use of gestural information to facilitate speech comprehension in adverse listening conditions. In line with previous literature, we expected a gestural enhancement effect for neurotypical adults (Drijvers & Özyürek, 2017). For autistic individuals, we expected a smaller gestural enhancement effect, based on the difficulties that have been observed in this group with processing both auditory (Ronconi et al., 2023) and visual (e.g., Silverman et al., 2010; Trujillo et al., 2021) information. However, some work suggested that these multisensory difficulties are overcome in adolescence (Beker et al., 2018; Foxe et al., 2015). We therefore expected some gestural enhancement of degraded speech comprehension to occur, albeit smaller than the effect we hypothesized for neurotypical adults.

## Methods

### Participants

Forty neurotypical (mean age = 24.1, *SD* = 4.17, 30 female) and 40 autistic individuals (mean age = 26.83, *SD* = 6.02, 26 female) participated in this experiment. All participants identified as White. Autistic participants were recruited via patient organizations, the Max Planck Institute for Psycholinguistics’ database and Radboud University. Autistic individuals held a clinical diagnosis. Specific data on socioeconomic status were not recorded. There were no differences in any of these demographic variables between groups.

### Stimuli

Participants were presented with 160 video clips of an actress who uttered an action verb accompanied by an iconic gesture or no gesture (see for a detailed description of pretests on recognizability and iconicity of the gestures, Drijvers & Özyürek, 2017), while the speech in the video was presented clearly or with 6-band noise-vocoding (see Drijvers & Özyürek, 2017).

### Experimental procedure

The experiment was conducted online using Gorilla Experiment Builder (www.gorilla.sc; Anwyl-Irvine et al., 2019). Participants were instructed to wear headphones. The experiment began with a sound check, to ensure a comfortable volume level and to verify that the participants were using headphones. Following these checks, participants completed questionnaires on demographics. In the main experiment, participants performed a free-recall task. Each trial commenced with a fixation cross (1000 ms), followed by the video (2000s ms). We asked participants to press the space bar as quickly as possible when they recognized the verb that was being conveyed by the actress in the video. After this, they could freely type which verb they thought was being conveyed. All videos were presented in a randomized order and divided over blocks of 40. Participants could take a self-paced rest after every block, and completed the experiment within 45 minutes.

### Analysis

For both groups, we calculated the Token Sort Ratio (TSR) for all responses. TSR uses fuzzy string matching to calculate the similarity between two strings (e.g., the correct answer and a participant’s answer), and returns a percentage overlap between 0 and 100. All responses that were slower than 4000 ms and faster than 300 ms (7.2% of the data, 920 entries) were excluded.

We then tested whether a gestural enhancement effect of degraded speech comprehension existed for both groups. To this end, we fitted linear mixed effects models (using the lme4 package, Bates et al., 2015) in *R* (version 4.2.3, R Core Team, 2023). P-values were obtained using the lmerTest package (v3.1.1., Kuznetsova et al., 2017). All models included a maximal random effects structure. When the model did not converge, we used the estimates of this model fit as starting values to restart the fit, and compared the estimates from different optimizers using the allFit() function. If these yielded similar values, warnings about singularity were considered false positives. This was the case for all model fits. To investigate whether the two groups differed in their gestural enhancement effect, we then ran another model including a three-way interaction between Speech, Gesture and Group, again using a maximal random effects structure.

### Community involvement statement

Our study was inspired by community members asking whether gestures could help or hinder communication in autistic people. Contact with these community members consisted of informal inquiries after other studies at our research center, at outreach events, and via social media (discussion boards, patient societies). We have discussed the design and results of this experiment with autistic people through two formal inquiries: one to a researcher on speech-gesture integration and one to a researcher on autism in language comprehension.

## Results

### Gestures enhance degraded speech comprehension for both neurotypical and autistic participants

Both neurotypical and autistic participants performed at ceiling in the clear speech condition, independent of gesture presence (see Table 1). When speech was degraded and gestures were present in the video, both neurotypical and autistic participants performed better than when speech was degraded and no gesture was present (see Table 1). Both groups demonstrated a gestural enhancement effect of degraded speech comprehension (Neurotypicals: *β* = 17.38, *SE* = 4.06, *t*(105.25) = 4.26, *p* = < .001; Autistic participants: *β* = 15.04, *SE* = 4.65, *t*(100.48) = 3.13, *p* = .001). The size of this effect did not differ between the two groups (*β* = 0.58, *SE* = 42.29, *t*(11640.49) = 0.11, *p* = .99; enhancement gain (%) in neurotypicals: 16.46%, enhancement gain (%) in autistic individuals: 14.63%, see for condition-specific percentages Table 1).

**Table 1.** Overview of accuracy results for neurotypical and autistic groups.

		CS/NG	CS/G	DS/NG	DS/G
Neurotypical	TSR (%)	99.00	97.83	64.17	80.63
	*SD* (%)	6.39	11.40	33.35	29.95
Autistic	TSR (%)	99.03	97.88	60.27	74.90
	*SD* (%)	6.16	10.87	36.27	34.97

TSR refers to the token-sort-ratio. CS/NG reflects clear speech, no gesture. CS/G reflects clear speech, gesture. DS refers to degraded speech. All numbers are presented in percentages.

## Discussion

In the current study, we asked whether a gestural enhancement effect of degraded speech comprehension exists in autistic individuals and whether this effect differed from the gestural enhancement effect that has been typically observed in neurotypical adults (Drijvers et al., 2019; Drijvers & Özyürek, 2017; Schubotz et al., 2021; Wilms et al., 2022). Our findings provide evidence that both groups benefited from the presence of gestures when speech was degraded and that this effect did not differ between groups. This suggests that iconic gestures can facilitate speech recognition in adverse listening conditions for autistic individuals, similar to what has been observed for neurotypical individuals. These results have important implications for understanding the role of multimodal signals in speech comprehension for autistic individuals. We discuss these results in more detail below.

We extended findings from previous work in neurotypical populations (Drijvers et al., 2019; Drijvers & Özyürek, 2017; Schubotz et al., 2021; Wilms et al., 2022) by demonstrating that autistic individuals show a significant enhancement in degraded speech comprehension when accompanied by iconic gestures. Contrary to our hypothesis that this effect may be reduced for autistic individuals due to their known difficulties in processing non-verbal information (e.g., Silverman et al., 2010), we found that this effect did not differ from neurotypical adults.

These results also do not concur with results from Silverman et al. (2010), who observed impaired gesture-speech integration in autistic individuals. Nonetheless, this divergence in results may be explained by differences in the experiment design: first, our sample included adult participants, whereas in Silverman et al. (2010) children and adolescents were included. As previous findings on visual speech (e.g., Foxe et al., 2015) suggested that multisensory integration difficulties in individuals ameliorate during adolescence and adulthood, the gestural enhancement effect that we have observed in our study may have developed with age. Second, the gestures used by Silverman et al. (2010) were redundant to the speech presented, whereas in our study gestures provided complementary information to speech. We therefore stipulate that a gestural enhancement effect in autistic individuals may be especially prevalent when there is a necessity to integrate stimuli coming from different modalities to understand speech, such as in adverse listening conditions. This would also fit with the Enhanced Perceptual Functioning theory (Mottron et al., 2006), if we assume that autistic individuals focus more on gestural information when speech is degraded, as gesture as a secondary/contextual cue becomes crucial for comprehension.

In the current work, we can exclude neither of these two options. Overall, our results extend what was previously reported by Silverman et al. (2010) to an older population and propose an interesting pattern, which should be tested and confirmed by future research: the presence of gestures facilitates speech comprehension in autistic individuals, whenever these gestures provide complementary information to speech, and this effect may develop with age.

### Limitations and future directions

Several limitations of the present study should be acknowledged. First, as discussed above, the current work only focused on autistic individuals of adult age. As previous work has suggested that for autistic individuals developmental differences may exist in multisensory integration (Beker et al., 2018; Foxe et al., 2015; Silverman et al., 2010), which ameliorate by early adulthood, future work could consider including different age groups to investigate whether similar effects occur for more complex visual information, such as the iconic gestures used in the current study. Second, the current work did not differentiate between different parts of the autistic spectrum, limiting the generalizability to all autistic adults. Third, we did not collect any data on the potential communicative difficulties that autistic individuals may experience during daily life. For example, autistic individuals may find it more difficult to filter relevant information from noise (Ronconi et al., 2023; Schelinski & von Kriegstein, 2020). Future work could investigate this by, for example, including questionnaires targeted at processing sound and speech in adverse listening conditions, or by including the actions and feelings questionnaire, which measures how well someone uses and understands visual communicative signals (van der Meer et al., 2022). Finally, the stimuli used in this work do not yet reflect the rich, multimodal context that we encounter in daily life. Although this study is a first step in that direction, these findings should be further replicated in ecologically valid circumstances, such as in richer sentence, or discourse contexts (see for example Matyjek et al., 2024; Wilms et al., 2022), using other parts of speech, or with other types of gestures than the iconic gestures used in this work.

## Conclusion

We here demonstrate that autistic individuals benefit from visual semantic information conveyed by gestures during degraded speech comprehension. This enhancement effect is not different from the enhancement effect that neurotypical adults experience and invite possibilities of incorporating this knowledge in interventions targeting (degraded) speech comprehension in autistic adults.
